# Barriers and prospects of India’s conditional cash transfer program to promote institutional delivery care: a qualitative analysis of the supply-side perspectives

**DOI:** 10.1186/s12913-018-2849-8

**Published:** 2018-01-25

**Authors:** Adyya Gupta, Jasmine Fledderjohann, Hanimi Reddy, V. R. Raman, David Stuckler, Sukumar Vellakkal

**Affiliations:** 1 School of Public Health, The University of Adelaide, Adelaide, South Australia Australia; 2 Department of Sociology, Lancaster University, Lancaster, UK; 3 Save the Children, Gurgaon, Haryana India; 4 WaterAid India, New Delhi, India; 5 Department of Policy Analysis and Public Management, University of Bocconi, Milan, Italy; 6 Department of Sociology, University of Oxford, Oxford, UK; 7 BITS Pilani Goa Campus, Zuarinagar, Goa 403726 India; 8 Public Health Foundation of India, Gurgaon, Haryana India

**Keywords:** National Health Mission, Janani Suraksha Yojana, Conditional cash transfer, Maternal healthcare, Institutional delivery care, Supply side perspectives, India

## Abstract

**Background:**

Under the National Health Mission (NHM) of India, Janani Suraksha Yojana (JSY) offers conditional cash transfer and support services to pregnant women to use institutional delivery care facilities. This study aims to understand community health workers’ (ASHAs) and program officials’ perceptions regarding barriers to and prospects for the uptake of facilities offered under the JSY.

**Methods:**

Fifty in-depth interviews of a purposively selected sample of ASHAs (*n* = 12), members of Village Health and Sanitation Committees (*n* = 11), and officials at different tiers of healthcare facilities (*n* = 27) were conducted in three Indian states. The data were analyzed thematically using ATLAS.ti software.

**Results:**

Although the JSY has triggered considerable advancement on the Indian maternal and child health front, there are several barriers to be resolved pertaining to i) delivering quality care at health-facility; ii) linkages between home and health-facility; and iii) the community/household context. At the facility level, respondents cited an inability to treat birth complications as a barrier to JSY uptake, resulting in referrals to other (mostly private) facilities. Despite increased investment in health infrastructure under the program, shortages in emergency obstetric-care facilities, specialists and staff, essential drugs, diagnostics, and necessary equipment persisted. Weaker linkages between various vertical (standalone) elements of maternal and primary healthcare programs, and nearly uniform resource allocation to all facilities irrespective of caseloads and actual need also constrained the provision of quality healthcare. Barriers affecting the linkages between home and facility arose mainly due to the mismatch between the multiple demands and the availability of transport facilities, especially in emergency situations. Regarding community/household context, several socio-cultural issues such as resistance towards the ASHA’s efforts of counselling, particularly from elderly family members, often adversely affected people’s decision to seek healthcare.

**Conclusion:**

Adequate interventions at the community level, capacity building for healthcare providers, and measures to address underlying structural and systemic barriers are needed to improve the uptake of institutional maternal healthcare.

## Background

The 3rd Sustainable Development Goal (SDG) targets ensuring good health and well-being for all at all ages by 2030, including reducing global maternal mortality and ending preventable child deaths [1]. Provision of sound quality of care at facilities during childbirth is globally advocated as a fundamental requirement for reducing maternal and child mortality [2]. Though maternal and child health (MCH) in India has improved over the past decades, these improvements have been slower than expected. At 45 deaths per 1000 live births for under-five child mortality [3], and 167 deaths per 100,000 live births for maternal mortality [4, 5], India ranks in the top 70 worst performing countries in both maternal and under-five child mortality [6].

In 2005, the Government of India [2] launched the National Rural Health Mission (NRHM) (revised to National Health Mission [NHM] since 2013), with an aim “to improve the availability of and access to free and quality healthcare, especially for people residing in remote areas” [7]. The JSY is a safe motherhood intervention scheme under the National Health Mission (NHM), and is an integrated program for addressing both supply of (improving infrastructure and quality of care) and demand for (getting women to facilities) facility-based skilled delivery care [8]. Together with strengthening the primary public healthcare infrastructure, there have been efforts to expedite resource allocation towards infrastructure investments. Towards this, the JSY also provides conditional cash transfers to women after delivery as per the program guidelines [9].

**Fig. 1:**
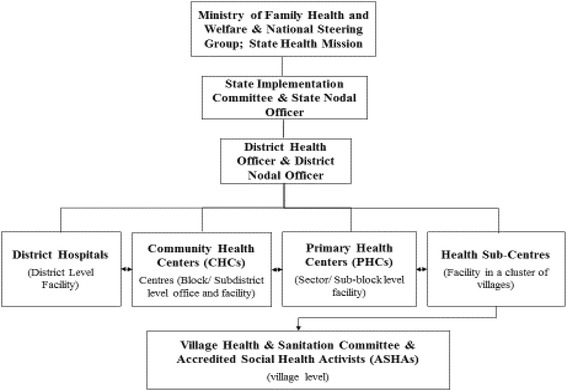
The JSY administrative hierarchy

Organizationally, a National Mission Steering Group oversees the overall implementation of the NHM, including the JSY. A visual representation of the JSY administrative levels is provided in Fig. 1. India’s four-tiered public health system deployed and supported a large force of around 1 million community health workers, named ‘Accredited Social Health Activists’ (ASHAs), in almost all rural villages of India [9–11]. On the ground level, the JSY is promoted by ASHAs, and supported by the Village Health and Sanitation Committees (VHSCs). Crucially, ASHAs help enroll pregnant women in the program, provide them with reproductive health knowledge and program information, and support them in accessing the program benefits through efforts such as accompanying women to delivery facilities for childbirth [9]. In Indian states with poor health outcomes (classified as ‘low-performing states’ under the NHM), ASHAs receive a performance-linked cash incentive for facilitating institutional delivery under the JSY- of INR 600 (US$9) in rural areas and INR 200 (US $3) in urban areas.

Emerging evidence suggests that institutional delivery is increasingly becoming a social norm across India due to the extensive promotion of the JSY and support offered by ASHAs [12, 13]. Nearly four-in-five women now deliver in health facilities, and more than one-in-three benefit from the JSY [14]. However, there exist substantial inter- and intra-state variations in the utilization of the JSY and uptake of facility-based childbirths. A UN report on the SDGs cites the JSY as a major contributor to improvements in MCH in India, but also points to the need for substantial scaling-up and restructuring of the program to increase efficiency and ensure continued success [15].

Most extant literature on the JSY has focused on the impact on MCH outcomes, and beneficiaries’ experiences of uptake of the services offered [16–21]. Previous research on the efficacy of the program has mostly focussed on (potential) beneficiaries, highlighting demand-side perspectives on the strengths and weaknesses of the program [12, 22–25]. There is a dearth of evidence on how the program is being implemented, particularly in terms of the program-related constraints, from a supply-side perspective. Moreover, though there is increasing evidence of poor quality of health systems in low- and middle-income countries (LMICs) such as India [26–28], little is known about the extent of improvement in the quality of health services at system levels, after the implementation of public health programs like JSY/NHM. Here, we documented perceptions of community health workers and program officials -- who either interact with people at grass-roots level or are directly involved in the implementation of the JSY-- regarding the enabling factors and barriers to the uptake of facilities offered under the JSY, and the measures (to be) undertaken to address them.

## Methods

### Study design and setting

In this cross-sectional study, we used qualitative data from in-depth face-to-face interviews. Interviews were conducted with participants at three levels: 1) community health workers, i.e. ASHAs and members of the VHSC; 2) intermediary level staff at Primary Health Centers (PHC) and Community Health Centers (CHC); and 3) higher level district officials, who are directly responsible for ensuring the provision of health services and monitoring of the program.

### Participants and sample selection

This study focuses on three populous, diverse ‘empowered action group’ (EAG) states of India: Jharkhand, Madhya Pradesh and Uttar Pradesh. The EAG states are characterised by poor performance on socioeconomic and healthcare indicators, and are targeted for additional interventions by several national social welfare policies. Among the 8 EAG states, Jharkhand has the lowest JSY uptake, Uttar Pradesh has a mid-range level of uptake, and Madhya Pradesh has the highest uptake.

Based on the level of economic development, two districts were purposively selected from each of these three states, with one being relatively more developed than the other (based on per-capita district income reported in the respective state development reports). Two blocks from each district were further selected purposively as the final sampling unit. In India, districts are administrative units within a state; within districts, there are smaller units, known as blocks or sub-districts. We aimed to recruit at least one district official from each of the selected districts. We then selected blocks from within the selected districts, and aimed to recruit one ASHA, one VHSC member, and one program official (i.e. planning and monitoring committee members) each from PHC and CHC within the each block. ASHAs were selected based on their availability (*n* = 12). Purposive sampling was also used to reach out to the district level officials (*n* = 6), program officials at PHC (*n* = 9) and at CHCs (*n* = 12), and members of VHSC (*n* = 11). A total of 50 interviews were conducted.

### Data collection

Data was collected over a period of 3 months from September to November 2013. We recruited a team of 5 male and 5 female interviewers with previous experience in conducting in-depth interviews for health-related research. The interviewers completed a 2-day training session (e.g. project overview, field practice) by the study team to familiarize them with the study context and ethical considerations. We provided the interview team with a semi-structured interview guide including some important probing questions prepared by the study team. Prior to the interviews, the interview guide was pilot tested on both ASHAs and district officials in the selected districts to assess its appropriateness, and changes were made wherever necessary. Any queries or differences that emerged during the process of pilot testing were resolved by the study team.

The interviews took place at the participant’s convenience; for instance, the program officials were interviewed in their offices. The interviews were carried out in the participant’s regional language (Hindi), and each interview lasted for 35-50 min. No one other than the participant and the interviewer was present in any of the interviews. Summary sheets were used to capture the demographic details of the participants. Probes were used wherever extraction of more relevant information was possible. All interviews were tape-recorded, and notes were taken during the interview, both to ensure that no important information was missed and as a failsafe against technological problems.

### Data analysis

Tape-recorded interviews were transcribed, and transcripts were verified with the recordings by the study team as a check on the quality and validity of the transcription. After transcription, the interviews were translated to English and back-translated to Hindi to verify the accuracy of the original translation. An inductive approach was used to identify patterns and themes [29]. Thematic data analysis was conducted using ATLAS.ti software. Transcripts were examined thoroughly by the authors (AG and SV), and topics that repeatedly emerged were highlighted and categorised as themes. These themes were reviewed and discussed between co-authors to avoid any personal bias, and to ensure analytic rigour. This was followed by further verification and validation of the themes with the available literature for the purpose of triangulation. The final themes were then summarized according to the pattern of findings (Table 1).

**Table 1:** Themes and subthemes categorized as barriers and remedial measures for India’s Janani Suraksha Yojana program

Themes	Subthemes	Categories
At the health facility level *(Receipt of adequate healthcare)*	Inability to manage birth complications Referrals to private institutions Insufficient healthcare personnel, medicine, equipment, distribution of resources Vertical program structures and standalone nature of MCH program components	Barriers
	Budgetary revisions to increase efficiency Greater oversight to ensure availability of equipment, medicine, and medical staff Employing more staff Introducing feedback and review system Building intra and inter program linkages	Remedial measures
Linkages between home and health-facility *(Timely transfer to health-facilities once the decision to seek health-facilities was reached)*	Limited availability of drivers and vehicles Delays in transport arrival High cost of alternative transport Poor quality of roads Inadequate understanding and poor attitude/ behavior of service providers Rent-seeking by service providers from beneficiaries	Barriers
	Provision of alternative transport Creation of an emergency number Regular monitoring of ambulances Budgetary revision to strengthen transport facilities Training, motivation building of service providers	Remedial measures
At the community and household level *(Decisions to use health-facilities)*	Decision-making power mostly influenced by elders in families Misconceptions about home vs. institutional birth Negative previous experiences ASHAs: inadequate support and capacities	Barriers
	Identifying pregnant women through direct observation and household visits Counselling services during prenatal, delivery, and ante-natal check-ups Awareness campaigns Counselling targeting male family members Utilizing media (e.g. radio, and TV) to provide reliable program information Strengthening ASHA support mechanisms	Remedial measures

## Results

The participants’ age ranged from 20 to 40 years for ASHAs and from 35 to 60 years for the district level officials and program officials. The ASHAs had all acquired education up to secondary and higher secondary level, with a few also holding bachelor’s degrees. Eighty percent of the program officials at PHC, CHC and members of VHSC were males with bachelor’s degrees.

Although the JSY has triggered considerable advancement on the Indian maternal health front (scaling up from 700,000 to 10.4 million beneficiaries and increasing expenditure from Rupees 38 to 1668 crore between 2005 to 2015) [30], there are several barriers to be resolved pertaining to i) delivering quality care services at the health-facility; ii) strengthening linkages between home and health-facility; and iii) tailoring services to the community/household context. These factors, in a reverse order, affected decisions to use health-facilities, timely transfer to health facility once the decision was reached, and receipt of adequate healthcare at the facility. We also identified the measures that were or could be undertaken to address these barriers, along with possible solutions for consolidating the gains of the program.

### Delivering quality care services at the health-facility

#### Barriers

Despite tremendous investment in health system infrastructure under the JSY/NHM [30], participants in our study felt the provision of adequate quality of care at the public health-facility level was constrained by the following broad range of dimensions: i) Inability to manage intra-partum and immediate post-partum complications, leading to increased referrals, mostly to private facilities; ii) insufficient healthcare personnel, medicine, infrastructure, and equipment; iii) uniform distribution of resources; and iv) the standalone nature of various elements of programs. Our results suggest that many of the systemic and structural barriers continue to exist, despite the considerable architectural correction introduced under the JSY/NHM. Participants saw these infrastructure-related issues as barriers to uptake of the program.

##### Inability of facilities to manage birth complications

All participants highlighted major gaps in preparedness of facilities and teams for providing adequate emergency obstetric care (EmOC) and the limited quality of care provided in emergency situations. The CHC and district officials highlighted that the limited availability of specialists was primarily due to scarcity, misdistribution, or absence of such specialists. They also pointed towards skill or confidence gaps to manage birth complications among available doctors and nurses leading to increased referrals, mostly to private facilities. The limited availability of specialized doctors and competent nurses was perceived to be the foremost reason for eligible women not seeking facility-based care. Even where specialized doctors and competent nurses were available, the support provided by health systems seemed inadequate, leading to depletion in their motivation and confidence for managing complications. Skill-building training, though held in many cases, was either inadequate or not appropriate to the specific needs of facilities—newly-trained personnel were often not deployed to where they were most needed. The ASHAs also often cited that many of the PHCs and CHCs, including some district facilities, were unprepared for managing complications related to childbirth, including provision of Skilled Birth Attendance (SBA) or Comprehensive EmOC. This was owing to insufficient availability or absence of skilled staff/well-equipped facilities. In order to ensure availability of staff and facilities, the NHM has mandated setting up First Referral Units (FRU) within an accessible distance for all localities. In order to qualify as an FRU, facilities must make round-the-clock services for EMoC and newborn care services available, in addition to all other emergency referral services that such a hospital is required to provide [31]. However, program officials noted that many of the designated CHCs and district hospitals failed to meet the FRU criteria in terms in of availability of services such as caesarean sections, laboratory tests, and blood transfusion or blood storage facilities. Moreover, those facilities which met (or nearly met) the service criteria did not meet the distance/access criterion for many of the localities in the districts.

“Doctors at government facilities can’t handle complications, and people are scared of death. Therefore they [patients] prefer to go to private hospitals for delivery.” (ASHA; Jharkhand)

##### Insufficient resources

The CHC officials highlighted that there were times when it was difficult to ensure 24-h availability of doctors and nurses at facilities. There is need for additional staffing at local facilities due to empty positions or vacancies, as there is scarcity of doctors and skilled nurses in rural and remote locations. Moreover, sometimes doctors went on short- and long-term leaves, with inadequate alternative arrangements during their absence - the facilities were in effect led by nurses in most cases, without the nurses having adequate support and authority to do so. Gaps in matching physician/nurse skills to facility needs, such as non-prioritization of specialist doctors or skilled nurses for facilities with a high caseload, further compounded the problem. For a few of the delivery points, CHCs and district hospitals in unison highlighted the problem of ‘overcrowding’, leading to compromise on issues like infection control, hygiene, immediate postpartum care, sharing of beds, duration of stay in facility following delivery, etc., despite high demand for services.

“The conditions of our hospitals are not good. We need manpower, and infrastructure for better results.” (District official; Jharkhand)

“There is lack of doctors and nurses. Often, doctors do not prefer to continue their service in rural settings for long time.” (Program official at CHC; Madhya Pradesh)

“Facilities like medicine, sanitation, hygiene and food provisions are poor and inadequate.” (VHSC member; Uttar Pradesh/Program official at CHC; Madhya Pradesh)

ASHAs noted that often families of pregnant women complained of poor quality of care in the health facilities. This included absence of basic healthcare facilities, such as a shortage of beds, electricity, water, toilet facilities, designated wards, essential medicines, diagnostics, advanced medical equipment, and emergency obstetric-care facilities. Gaps in the availability of well-trained nurses and specialist doctors to treat complications added to this.

“We tell people about of the benefits of going to hospitals for childbirth, but people tell me that in hospital there is no doctors or nurse and even beds and electricity, so why should we go there.” (ASHA; Jharkhand)

“In every PHC there should be at least 2-3 properly trained staff. Maternity ward should be constructed for deliveries.” (District level official; Madhya Pradesh)

However, the staff members of the health facilities argued that their efforts relied critically on the willingness of doctors to serve in the rural settings, timely release of funds, and an adequate supply of medical equipment and medicine from the state level. The higher level officials felt that they were taking action to address infrastructure and staffing complaints, but were constrained by factors outside of their control.

“Less doctors and nurses apply for positions here because they get less remuneration, and they don’t want to serve in this kind of rural settings. State should allocate more funds to keep more staff that can manage complications.” (Program official at CHC; Uttar Pradesh)

##### Uniform distribution of resources

Most of the intermediary level officials cited a mismatch between human resources, equipment/technology, and physical infrastructure availability across the facilities, which they felt resulted in suboptimal service provision. Overall, the available supplies and resources were distributed uniformly across all the facilities without attention to the actual caseload demands, infrastructural needs, or utility of these supplies within specific facilities. This ‘equal’ rather than ‘equitable’ distribution approach hindered efficient and effective functioning of some facilities where the need for resources was higher, while also resulting in wastage of resources in other facilities. The operational processes in the health systems lacked the concept of redistribution (redistribution was partly but not meaningfully applied in the case of human resources through transfer processes), leading to the continuous occurrence of these otherwise preventable wastages.

“There is ongoing problem of inadequate funds and essential services at the facility which is a persisting concern.” (Program official at PHC; Uttar Pradesh).

According to ASHAs, the conditional cash incentive given to them against the task was much lower than the expenditure they incurred while helping pregnant women to access facility-based care.

“Our allowance should be increased. It is hard to perform our duties with less money.” (ASHA; Uttar Pradesh)

##### Vertical integration of various program elements

Another important concern, primarily reported by higher level officials, was about the standalone nature of the program structures for various MCH initiatives, including the JSY. Additionally, continuous changing of health priorities of district and state administration, leading health workers to perform according to instructions from higher level, was a systematic problem highlighted by the officials. It was reported that most components of public health programs lacked essential integration and coordination with various other specific programs related to MCH and overall health such as i) family planning, ii) child immunization, iii) adolescent health, and iv) prevention and control of several life-threatening diseases. Although the NRHM (the precursor to the NHM) recognized this problem and revised the program design to integrate reproductive and child health (RCH) as its key component, continued partitioned funding and technical support have in effect kept the RCH (and the other components) functioning almost as vertical programs. Specifically regarding the JSY, the NRHM mission documents very clearly identify the JSY as its flagship program for improving quality of care and hence the maternal health outcomes. Yet officials lacked clarity as to whether the JSY was a program to improve quality and outcomes of MCH services or it was simply a cash incentive program to shift childbirths to the facilities. Most believed the latter, and failed to realize its strong linkages with quality of MCH services, and the program’s potential to improve these services. Such perceptions also seem to have isolated the JSY from the overall functional ambit of RCH and the broader program mission.

“This program may be more effective if it is integrated with other programs under MCH as it will help to overcome several barriers, especially funding.” (District official; Madhya Pradesh)

#### Remedial measures

District officials reported that they regularly monitored monetary inflow and outflow in their district, and strategically planned for both securing and spending funds as appropriate. Efforts were made for budgetary revisions, timely release of funds, additional provisions of food and medicine to expectant mothers, infrastructural changes, and improved staffing. They further reported that expenditures were meticulously budgeted for activities relating to improving hospital infrastructure and stocking medicine. To address many of the issues brought up by the ASHAs, ‘Sahiyya’ Help Desks (a support mechanism for ASHAs) were set up in some hospitals in Jharkhand.

“Yes, we have a monitoring team and we plan the allocation of funds for different purposes.” (District official; Jharkhand)

Other suggested measures for improvements in the provision of care at the facilities include adequate finances and human resources for fulfilling all requirements that are needed for attaining FRU status in select facilities. Also suggested were creation of adequate systems for staff training and motivation, and systemic interventions such as regular and transparent review and feedback mechanisms to identify and address various institutional and operational constraints. Policy level interventions such as need-based posting of specialists and skilled nurses, improved implementation of EmOC or Basic EmOC centres, and strengthening skilled-birth attendants’ training were also identified. Differential approaches in financing, staffing, and equipping the facility based on the actual caseload of each facility was also suggested as an important way forward.

### Linkage between home and facility

#### Barriers

The linkage between home and facility was constrained by two major factors-- transport services and the perceived quality of services at the facility.

##### Transport services

A patient’s inability to reach a health facility in time for delivery was described unanimously by respondents as a barrier to the uptake of health services. This was mostly due to the poor availability of transport facilities, especially a shortage of drivers/ambulance staff. The intermediary level officials reported that the state government has been providing free ambulance facilities to pregnant women to reach health facilities. However, the limited or non-availability of drivers, especially during nights and holidays, and a mismatch between unexpected multiple demands for a limited number of ambulances often constrained the program efforts. Some officials thought that the ambulances were sometimes used as general transport for department purposes, thereby reducing their availability for pregnant women to access facilities. Furthermore, damaged and inaccessible roads in remote areas made it difficult for the ambulances to reach women in time. ASHAs recalled that there were times when the government ambulance was either unavailable or took longer than expected to arrive at the residence of the pregnant women and they had to rely on other modes of transport- often private services with higher charges, or even non-conventional transport. This sometimes also resulted in deliveries taking place at home under the supervision of unskilled birth-attendants (someone with no prior training in conducting birth delivery, such as ‘dai’, the traditional birth attendant) or sometimes under the supervision of ASHAs themselves.

“There is less number of ambulances and drivers. It’s difficult to get the driver in night and early morning.” (Program official at PHC; Uttar Pradesh)

“The roads are damaged and it is difficult to reach to hospitals in time.” (ASHA; Jharkhand)

##### Perceived quality of services at facility

Several factors related to pregnant women’s perceptions around the overall quality of care, including the behavior of the care providers, affected the linkage between home and facility. In addition to major issues related to the quality of care (discussed above), often the rude behavior and the rent-seeking (bribes/side-payments) of the care providers from patients and ASHAs discouraged patients from using the facility. Overall lack of cleanliness and hygiene in the facilities was another issue that was reported.

“Sometimes the staff at the hospitals asks for money from the patients after delivery. This makes it difficult for us [ASHA] to convince the patients towards facility-based delivery.” (ASHA; Jharkhand) “Hospitals are far away, it may bring financial hardship for poor to arrange a vehicle.” (ASHA; Madhya Pradesh)

#### Remedial measures

To ensure the timely availability of transport facilities, the intermediary program officials at PHCs and CHCs mentioned that they regularly monitored the availability and functionality of ambulances and their drivers. During periods of limited availability of the ambulance, they explored other options to ensure that alternative vehicles reached pregnant women in time. ASHAs also provided telephone numbers to pregnant women for use during emergency situations, and escorted them to the facility.

“We give advice, and try to arrange for vehicle when ambulance is unavailable.” (Program official at CHC; Madhya Pradesh)

“We escort women for the delivery to hospital and also while coming back from the hospital.” (ASHA; Jharkhand)

Better planning to ensure timely arrival of vehicles, improving the management of ambulance services, and identifying need-based alternative transport services were suggested by ASHA’s. ASHAs further argued that the expense incurred in organizing alternative vehicles should be reimbursed to those who organized such arrangements, whether they are ASHAs or the families of pregnant women. This would help all parties avoid any monetary loss or burden. It was mentioned that charges for such alternative transport to carry pregnant women in emergencies were always much higher than the market rates. There were suggestions towards regulating this conditional high pricing as well.

“Government should provide more number of ambulances and employ more drivers to be available during emergencies and night times.” (ASHA; Uttar Pradesh)

“Alternate vehicles charge lot of money to go to the hospital, we [ASHA] should either not be charged or get back all the money we [ASHA] spend to take the patients to the hospital.” (ASHA; Jharkhand)

In order to improve the behavior of health facility staff towards mothers and ASHAs, it was suggested to focus on better training and capacity building, regular performance review, and rectification measures. Increasing clarity about ASHA’s roles and their importance in health services, and ensuring that health service providers display more respect towards them were listed as important measures.

“Key to improving the facility uptake is to improve the performance of the staff by providing them adequate training. Also, regular actions should be taken to resolve any kind of issues arising.” (District official; Madhya Pradesh)

“Allocate more budgets for ambulance and drivers as they are important.” (Program official at CHC; Uttarakhand)

“We think that without changing anything within the program of the JSY, we should bring changes in its techniques. For example improvement in escort service by community health workers [ASHA] will ultimately help reduce maternal and child mortality.” (District official; Uttar Pradesh)

### Community/household contextual factors

#### Barriers

Decisions to seek care were reportedly influenced by several factors embedded in the social and cultural milieu. According to ASHAs, lack of awareness about the importance of medical care during childbirth, combined with preconceived beliefs of elderly people in favor of home-based childbirth, were jointly associated with a reduced uptake of the JSY. In some cases, ASHAs were unable to effectively provide counsel on the benefits of MCH services to persuade people to overcome their rigid stance against facility-based childbirth. There were instances of resistance against the counseling services of ASHAs, particularly from elderly family members.

“People are not considering the benefit of the JSY, some of them decide about the place of childbirth according to their family.” (ASHA; Jharkhand)

“Some people are not using the JSY services because of their own thoughts. We try to convince such kind of people but still some people are stubborn they don’t understand it despite the problems.” (ASHA; Uttar Pradesh)

While these constraints were mainly reported by ASHAs, both the intermediary and higher levels officials concurred.

“Some people don’t understand the benefits of going to hospitals for delivery.” (Program official at CHC; Madhya Pradesh)

#### Remedial measures

ASHAs reported that they took several steps to positively influence the decision to seek care. This included identifying women at an early stage of pregnancy through direct observation and speaking to relatives in order to establish a relationship, followed by individually-focused counseling to potential beneficiaries on a wide variety of MCH issues.

“First we ask family members like mothers-in-law of the women and sometime we identify pregnant women just by observing them.” (ASHA; Jharkhand)

As an additional strategy to foster wider outreach of the JSY, ASHAs and VHSC members reported that they jointly conducted several awareness initiatives highlighting the benefits of the program and the facility-based childbirth. Health department officials were in agreement with this as well.

“We, doctors and health committee collectively make (conducts) awareness campaign (s), ‘nukkad naataks’ [street-show] and spread posters across the district to help make youngsters aware of the program.” (ASHA; Uttar Pradesh/District official; Jharkhand)

Officials and ASHAs suggested increasing the number of ASHAs household visits. Further suggestions were made for wider use of public broadcasting through television, radio, and newspapers for the dissemination of information on the messages regarding the benefits of the JSY and MCH services beyond cash incentives. It was felt that such modes of information dissemination would further facilitate ASHAs’ efforts of counseling and support services because this would enable the potential beneficiaries to easily relate ASHAs services to the larger framework of the maternal safety and child survival program.

“Apart from us [ASHA], if people get information from TV and radio it will add value to our counseling and all efforts we make.” (ASHA; Madhya Pradesh)

Improving the support measures for ASHAs has been suggested for increasing the effectiveness of ASHAs services, and for establishing their identity as acceptable counsellors on health-related issues. Suggestions were made for setting up permanent support systems for ASHAs that includes on-site support in problem solving by supervisors, regular platforms to enhance their motivation, ongoing training and capacity building, addressing various challenges that they face with communities and at health/related departments, and inculcation of a respectful functional environment for them at both the community and facility levels. A further suggestion was made to build a clearer link between the ASHA and the facility in the delivery ward, as often the mothers who accompany ASHAs to the facility expect a level of moral and emotional support from the ASHA at the time of delivery. However, due to a missing linkage between facility staff nurses/medical officers and the ASHA, often ASHAs are sidelined at the facility and feel let down in front of the family who opted for facility on her advice. This missing linkage is more visible in facilities with high caseloads. VHSC needs such regular institutionalized support too, in order to optimize their ability to influence the uptake of MCH and other health services and programs.

“Training programs to support and guide ASHAs should be organized on a regular basis to strengthen their interest and motivation towards empowering the community.” (Program official at CHC; Uttar Pradesh)

### Contrasting views of participants emerged regarding program infrastructure

Though the state governments offer free transport facilities to patients for timely access to facilities, still there is a mismatch between availability and demand. The higher level officials reported on their monitoring the availability of ambulances and efforts to ensure that alternative sources of transport were available where needed, but ASHAs reported from the ground level that there was a continued shortage of ambulance and alternative transport facilities. This apparent disconnect between ASHAs and district-level monitoring committee reports may be explained by budgetary and management factors.

“We try to provide the facility of ambulance by regularly monitoring its availability and also provide alternate vehicles in the absence of ambulances.” (Program official at PHC, Ghaziabad, Uttar Pradesh)

“There is lack of number of ambulances available and it is extremely difficult to arrange an alternate vehicle especially during night hours.” (ASHA; Uttar Pradesh/District official; Jharkhand)

#### Data not used for decision-making

On a monthly basis, ASHAs and Auxiliary nurse midwife (ANMs) supply their performance data, including JSY beneficiaries, to their respective PHC, and PHCs in turn supply this data to the district as part of the management information system (MIS). One of the cross-cutting barriers highlighted by district officials, however, was that this data is rarely and inadequately analysed at district level. In the absence of such an analysis, programmatic decision-making, both at the district and lower levels, is mostly done on an ad-hoc basis without incorporating important insights from real-time data.

## Discussion

The major themes that emerged from our analysis were consonant with the analytical frame of the ‘3 delay model’ proposed by Thaddeus and Maine [32, 33]. The delay model identifies three domains of delays that could affect healthcare access: (i) the decision to seek appropriate medical help, (ii) reaching an appropriate health facility, and iii) receiving adequate quality of care at the facility. We found that implementation barriers under the JSY fell into these domains, where socio-cultural barriers at the community/household level led to delay in the decision to seek appropriate care; infrastructural barriers in linkages between home and the facility led to delays in reaching healthcare facilities; and health system barriers often compromised the provision of quality care at the facilities.

A key finding of the current study is that the provision of quality care is often constrained by health system-related barriers consisting--inadequacy of personnel and non-personnel resources--consonant with other studies [23, 34, 35]. The current study highlighted critical supply-side barriers to the JSY uptake, including the shortage of specialist doctors and infrastructure to treat birth complications, vertical elements of maternal and primary healthcare, and the almost uniform allocation of resources across facilities. We also found that gaps in institutional measures to ensure proper understanding and implementation of the JSY have led to a widespread interpretation of the JSY as a simple cash transfer scheme; yet the institutions that ensure improved quality and outcomes of maternal health services are crucial for making *Janani Suraksha* (meaning maternal safety in Hindi) a reality. The program, through a supply-side lens, seems to remain primarily a promotional scheme to shift the place of childbirth to healthcare facilities rather than an organic program to improve the quality and security of childbirth processes. Policy attention needs to be directed to attending these hard realities.

The contrasting views between ASHAs and district-level monitoring committees on infrastructural barriers were also reported in other studies, which highlighted that often people living in remote locations are not well-connected to major roads, and face challenges in reaching hospitals due to long distances and/or unavailability of transport [12, 36, 37]. ASHAs in these areas may have a better sense of the accessibility challenges, and their perspectives provide an important resource for identifying and addressing context-specific resource needs.

Among the socio-cultural barriers embedded at the community/household level, misconceptions about institutional delivery and resistance to the counselling services from elderly family members were highlighted as key challenges. This is in keeping with other studies that report the influence of similar socio-cultural barriers on individuals’ health-seeking behaviour [16, 36, 38–40]. Informed decision-making is a prerequisite to improved uptake of services. Lessons from other states in India with low maternal and child mortality rates like Kerala and Tamil Nadu should be harnessed. In these settings, family education (as opposed to a focus exclusively on maternal education) played a key role in improving health outcomes and increasing the use of health services [41].

In India, despite the reported positive impacts of the JSY (for instance, reorganisation of physical infrastructure that led to increased uptake in Rajasthan, critical implementation issues persists. Regional imbalances in health infrastructure endure, with high-focus states lagging behind in implementation as compared to low-focus states [17, 42]. A multi-state study in India reports insufficient training of community health workers, staff absenteeism, and apathy of doctors posted in rural areas as some of the key health system constraints [43]. In India, a preference for private facilities continues to exist; findings from our study suggest that frequent referrals to private facilities may compound this problem by eroding faith in the public system. Some evidence suggests, however, that appropriate staff behaviour and the availability and good physical infrastructure of the facility can help to overcome this preference [36]. Higher levels of patient dissatisfaction are often a result of staff non-attendance, lack of medicine, long waiting times, and inability of staff to manage complications [44].

### Policy implications

Our study gives insights towards strengthening supply side infrastructure and interventions in order to improve services. Better maternal health outcomes will mostly depend on supply-side improvements, both in terms of perception and action. The Government of India initiated several measures to address supply-side gaps beginning in the early 1990’s [31]. Despite this, the JSY/NHM in general and across several states, including the 3 states in this study, is struggling to address these ongoing infrastructural challenges. Importantly, the state of Tamil Nadu has made ‘Comprehensive EmOC’ available in all parts of the state, within 30-40 km of travel for the users; however, such services are distant dreams for most of the remaining states due to the constraints outlined in the study. While most states planned to develop 3-4 FRUs for MCH per district, such plans are yet to be translated to reality in many cases. Focus needs to be placed on achieving these goals as soon as possible. The key area for improvement is ensuring the availability of specialist doctors and state-of-the-art infrastructure to treat birth complications, at least in the district hospitals and the designated FRUs, EmOC centres and Basic EmOC centres. An alternative approach of short-term training for doctors in specialist skills has been accepted as a solution, but needs to be better-implemented.

Given the current program environment, a scale-up of ASHAs services [45] may open new avenues for improvement in MCH uptake. ASHAs can serve as effective intermediaries between the community and the healthcare system for improved healthcare access, and can also provide a unique perspective on ground level barriers (such as rural road infrastructure) that may be less visible to higher-level authorities. Continuous support to ASHAs in the form of regular training for capacity building and motivation is required, in addition to fully covering all program-associated costs, including the livelihood loss that ASHAs incur.

Furthermore, our findings suggest that resource allocation to each facility should be based on the caseload and actual needs, replacing the current strategy of equal allocation across facilities; inaccessible and underprivileged areas may need bespoke, targeted strategies. An integrated approach of effectively combining elements of various national and state government level programs for greater efficacy and outreach of the MCH services is needed. Lessons can also be drawn from the experiences of other states, such as Tamil Nadu and Kerala, where a strong political commitment of the state government towards family planning, sustained information programs, increased education, and an increase in the standard of living have led to high performance on a range of MCH indicators.

### Strengths and limitations of the study

A major strength of this study lies in the richness of our data, collected across a range of health workers and program officials. The findings of this study are generalizable to at least the EAG states in India, as we purposively selected one state each from low-, medium-, and high-performing states. Further, considering that the barriers we identified are central to the program, similar barriers may co-exist across all states in India. The cross-cutting perspectives supplied by the informants on the preparedness and the constraints faced by the JSY provided an in-depth overview of the strengths and weaknesses of the current system. This study explored the perspectives of the stakeholders, who are directly involved with the implementation of the scheme at the grass-roots and intermediary levels--a highly relevant but often a missing perspective in policy discussions.

One of the limitations of this study is the extent to which the health officials may be reluctant to report on problems in the system; response bias among participants could feasibly have resulted in under-reporting of health system barriers and over-reporting of their personal efforts. However, given their integral roles in ensuring the smooth functioning of the JSY, these stakeholders are best-placed to illustrate on the strengths and weaknesses of the program from a health system perspective.

## Conclusion

With institutional deliveries slowly becoming a societal norm in India, quality of intrapartum and immediate postpartum services is the need of the hour for sustaining JSY program use, and for mitigating out-of-pocket expenditures to families for delivery care. Our findings suggest that some health system gaps continue to exist. In order to enhance the effectiveness and efficiency of the program and the health system, it is vital to focus on possible ways to improve the supply side interventions- allocating human resources and supplies, planning for equipping facilities, improving provider attitudes, strengthening linkages between facilities and community, and increasing the quality of care - in addition to tackling underpinning socio-cultural barriers. A more comprehensive system involving equitable resource allocation and management and coordination of functions and responsibilities across different levels of stakeholders would be beneficial in improving overall uptake of the program, thereby improving MCH services. Preventable maternal and neonatal deaths are not restricted to home births; poorly equipped facilities, lack of availability of well-trained specialist staff, and limited resources all contribute to poor MCH outcomes at the facility level. An improvement in these services therefore also has the potential to reduce both maternal and neonatal mortality.

## Abbreviations

ANM: Auxiliary nurse midwife
ASHA: Accredited social health activists
CHC: Community health centre
EAG: Empowered action group
EmOC: Emergency obstetric care
FRU: First Referral Unit
INR: Indian rupee
JSY: Janani Suraksha Yojana
LMIC: Low and middle income countries
MCH: Maternal and child health
MIS: Management information system
NHM: National Health Mission
NRHM: National Rural Health Mission
PHC: Primary health centre
RCH: Reproductive and child health
SBA: Skilled birth attendance
SDG: Sustainable Development Goal
UN: United Nations
US$: United States dollar
VHSC: Village Health and Sanitation Committees
